# Stereo Vision-Based Underground Muck Pile Detection for Autonomous LHD Bucket Loading

**DOI:** 10.3390/s25175241

**Published:** 2025-08-23

**Authors:** Emilia Hennen, Adam Pekarski, Violetta Storoschewich, Elisabeth Clausen

**Affiliations:** Institute for Advanced Mining Technologies (AMT), RWTH Aachen University, 52062 Aachen, Germany; apekarski@amt.rwth-aachen.de (A.P.); vstoroschewich@amt.rwth-aachen.de (V.S.); eclausen@amt.rwth-aachen.de (E.C.)

**Keywords:** stereo vision, automation, underground mining, environmental perception, LHD

## Abstract

To increase the safety and efficiency of underground mining processes, it is important to advance automation. An important part of that is to achieve autonomous material loading using load–haul–dump (LHD) machines. To be able to autonomously load material from a muck pile, it is crucial to first detect and characterize it in terms of spatial configuration and geometry. Currently, the technologies available on the market that do not require an operator at the stope are only applicable in specific mine layouts or use 2D camera images of the surroundings that can be observed from a control room for teleoperation. However, due to missing depth information, estimating distances is difficult. This work presents a novel approach to muck pile detection developed as part of the EU-funded Next Generation Carbon Neutral Pilots for Smart Intelligent Mining Systems (NEXGEN SIMS) project. It uses a stereo camera mounted on an LHD to gather three-dimensional data of the surroundings. By applying a topological algorithm, a muck pile can be located and its overall shape determined. This system can detect and segment muck piles while driving towards them at full speed. The detected position and shape of the muck pile can then be used to determine an optimal attack point for the machine. This sensor solution was then integrated into a complete system for autonomous loading with an LHD. In two different underground mines, it was tested and demonstrated that the machines were able to reliably load material without human intervention.

## 1. Introduction

Load–haul–dump (LHD) machines are commonly used in underground mines for short-distance material transport. These rubber-tired, articulated machines load material from muck piles in working faces and haul it to dumping locations. The first type of this machine was invented by Wagner at the end of the 1950s [1,2]. According to Tatiya [3], nowadays, over 75% of the world’s underground metal mines utilise LHDs. As every mine is unique, to address the specific customer needs, LHDs are available on the market with various bucket sizes, designs, and features.

Automation plays a major role in increasing operator safety and boosting productivity. There are already solutions with modern LHDs that offer autonomous hauling and dumping [2] at fixed locations, such as an orepass. The LHD is monitored by an operator from a remote control room, who can take over control if necessary. A bottleneck is the bucket-loading process, which is, in general, not fully automated and, therefore, carried out remotely by an operator. But LHD manufacturers are working on changing this. For example, in June 2023, Sandvik from Sweden launched a new feature called AutoMine^®^ AutoLoad 2.0 that enables operators to instruct LHDs on different loading profiles tailored to every drawpoint, thereby eliminating the necessity of an operator’s assistance when loading from fixed drawpoints [4]. However, this approach requires the LHD to be trained first, or existing loading profiles must be selected by the operator before loading from a new drawpoint/location. In addition, it is limited to fixed drawpoints that usually have a constant point of attack or at least not much room to vary the attack point.

To reach fully autonomous bucket-loading, the first challenge to be tackled is autonomously detecting the muck pile to be loaded and its relevant characteristics. The information about the position and shape of the muck pile can then be used to determine the point of attack. This eliminates the need to teach the machine different loading profiles, and a higher level of autonomy can be achieved.

For sensor-based muck pile detection, the following requirements must be fulfilled:

- The decision of which part of the muck pile to load is made during the drive. Therefore, the detection must take place while driving, as well as from a considerable distance. If the detection were performed from a close distance, the LHD would need to reverse to be able to drive into the muck pile. The resulting loss of time and, consequently, the loss of productivity must be prevented. In the specific application considered in this work, a minimum distance of 15 m between the LHD and the muck pile was specified by LHD manufacturer Epiroc.
- The muck pile needs to be differentiated from the drift walls and floor, which consist of the same material. Otherwise, there is a risk of collision and damage to the machine.
- Changes in the size and shape of the muck pile, as well as rock fragmentation, must be possible. Moreover, the system must be capable of detecting an empty working face to stop the loading operation and avoid machine damage.
- Detection must be possible under varying, even harsh operational (vibration and shocks) and environmental conditions (dust, humidity, and a wide temperature range).
- Muck pile detection must be guaranteed without additional light sources in the working faces.

This paper presents a solution for stereo vision-based muck pile detection for autonomous LHD bucket loading, which was developed as part of the Next Generation Carbon Neutral Pilots for Smart Intelligent Mining Systems (NEXGEN SIMS) project funded by the European Union’s Horizon 2020 research and innovation program. One focus area of the NEXGEN SIMS project was the autonomous material handling, comprising the autonomous loading of the muck pile with an LHD and unloading into an underground mine truck without the intervention of humans [5]. Firstly, related work on muck pile detection in a mining environment is addressed. The novel stereo-based approach is then described, followed by test applications in two different underground mines. The paper ends with an outlook on future work.

## 2. Related Work

Table 1 shows an overview of previous works tackling the problem of muck pile detection in a mining environment. While many of these works also present methods beyond this particular problem, to look at the loading process more broadly here, only the muck pile detection itself is considered, as this is the most relevant to this work.

Back in the early 2000s, Whitehorn et al. [6] utilised a stereo camera to perceive the environment around an LHD in an underground mine. The resulting point clouds were integrated over time to build a digital elevation model of the area and localize the LHD inside this map. However, no detection of specific structures like muck piles took place.

Sarata et al. [7] used a stereo camera to observe the environment and build a column model from the resulting point cloud. The pile edges were assumed to be where there are columns in a specified height range above ground. This implicitly assumed a free-standing muck pile on flat ground and was applied above ground. Its precise edge was determined with a laser range finder.

Magnusson and Almqvist [8] obtained a point cloud of the environment with an actuated lidar. The system then first determined the eigenvectors and eigenvalues of the points around a given point. To classify this point as belonging to a pile, the surface had to be relatively flat (meaning there had to be one dominant eigenvalue) and the right inclination. Then, nearby points were clustered together and segmented to obtain the final pile detection. The method was tested on simulated data and on a gravel pile, which was stationarily scanned from different positions. It is unclear how well this method would work when applied from a moving vehicle, especially as the complete computation time for a single scan was not given.

Backman et al. [9] simulated a narrow drift to perform reinforcement learning in order to determine an optimal attack point. The simulated sensor is a depth camera installed in the stope. Explicit pile detection was not performed, but it had to occur implicitly through the machine learning model to determine an attack point. An attack point was always generated, so the question of whether a muck pile is even visible is sidestepped in this setup.

Tampier et al. [10] combined scans from two 2D laser scanners installed on an LHD machine and used odometry to merge single frames into a complete 3D point cloud. The pile inside this point cloud was then detected based on the normal vectors of the local surface around the points: when the normal vectors point along the y-axis, the point is assumed to belong to the walls. After some additional filters were applied, the slope of the remaining pile was determined, also based on normal vectors, and the pile was considered detected when it was neither too flat nor too steep to be safely loaded. The pile was localized, but the geometric form was not considered here, as the system was specifically designed for narrow drifts typical in sublevel stoping, for which determining an attack point does not make much sense, as the LHD does not have enough room to navigate. The goal was simply to hit the pile while avoiding collisions with the surrounding walls. If the muck pile detection was tested separately, it is unclear from the work.

Cardenas et al. [11] built on the work of [10] by applying the system to a room-and-pillar mine and modifying it so that it could handle a new environment with wider tunnels where attack points have to be selected to load from a pile evenly. Among other things, the pile detection had been modified to reliably determine its geometric shape and contrast it from the ground. Also, the tunnel geometry was considered to determine whether the machine had enough space to maneuver. From the description, it is unclear whether the muck pile detection is performed while driving at normal speed towards the muck pile or whether the LHD first comes to a stop so that the muck pile detection can be performed from a stationary viewpoint. The distance from which a muck pile is usually detected is also unclear from the experimental results.

Jari and Lauri [12] presented AutoLoad 2.0, a system for automatic loading with an LHD. It uses predefined routes on which the LHD navigates. Lidar sensors are used to avoid collisions with the walls. Entering into a muck pile is detected via clutch slip. With this setup designed for fixed drawpoints and narrow drifts, no explicit muck pile detection is needed. This autonomous loading solution, called AutoMine^®^ AutoLoad2.0, was launched by Sandvik on the market in 2023 [4].

It is unclear whether a previous work performed muck pile detection while driving at normal speed and from large enough distances that the machine can drive towards the attack point without slowing down or even reversing. Achieving this goal would be an important step towards an efficient autonomous loading process. Beyond that, to the best of our knowledge, no previous work used a stereo camera for underground muck pile detection. Compared to a lidar sensor, it provides visual information linked with the computed 3D point cloud, which can be useful for subsequent detection tasks down the line and which makes achieving muck pile detection with a stereo camera a necessary requirement.

## 3. Novel Approach to Muck Pile Detection

**Figure 1:**
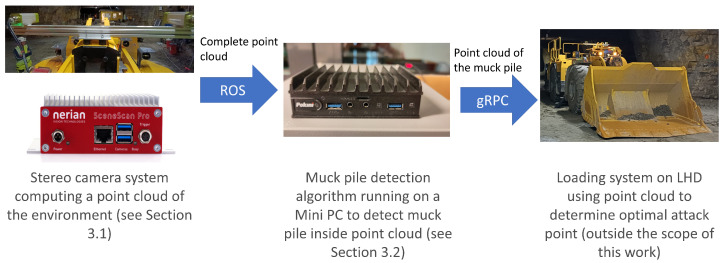
Components of the muck pile detection system.

Our proposed method is based on a stereo camera mounted on an LHD. It continuously creates images and computes a point cloud from them. In the next step, this point cloud is further processed using a topological algorithm to decide whether it contains a muck pile and extract it. This information is sent to an internal software system controlling the LHD. This system will use the information to determine a good attack point and navigate to this point. The main components of the setup are illustrated in Figure 1.

### 3.1. Stereo Vision from an LHD

For the stereo camera system, two monochrome cameras were chosen. Monochrome cameras produce grayscale images. As each pixel captures the entire incident light, they have a greater sensitivity to light compared to color cameras [13]. In poor lighting conditions, such as those found in underground mines, better image results can be achieved. Color information is unimportant for muck pile detection, as the surrounding area usually consists of the same material. Also, higher frame rates and shorter processing times can be realized with monochrome cameras [13].

The stereo camera system used consists of two monochrome cameras mounted on a profile with a baseline distance of approx. 1 m. The stereo camera was then mounted on a sensor platform on top of the LHD, around 5 m behind the bucket. The relatively large baseline allows accurate measurements from a greater distance. Usually, such a large baseline involves the drawback of objects close to the cameras only being seen through one camera, and therefore, no depth can be estimated. In our case, however, this is actually an advantage, as the first few meters before the cameras will mostly show the LHD itself, which is irrelevant for detection tasks. Keeping parts of the LHD out of the point cloud reduces clutter and increases focus on the more relevant parts of the camera images.

To assist the cameras, additional headlights were installed on the machine. Our experiments showed that, while they are not necessary to detect the muck piles, the additional lights both improve the detection quality and enable the system to work from greater distances, which in turn gives the LHD more time to navigate to the selected attack point.

The cameras were placed in industrial enclosures (IP66/67) to protect them from dirt and water (both powerful water jets and immersion up to 1 m). In order to secure the cameras from vibrations and shocks during operation, vibration dampers were attached to the mount.

**Figure 2:**
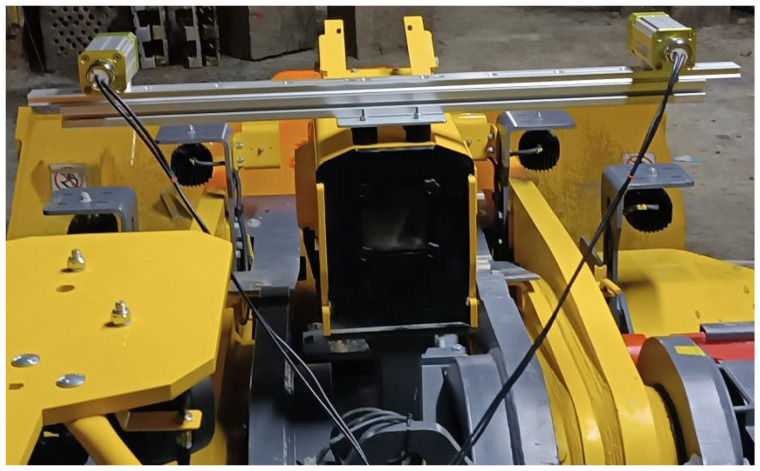
Camera installation.

The camera installation can be seen in Figure 2.

**Figure 3:**
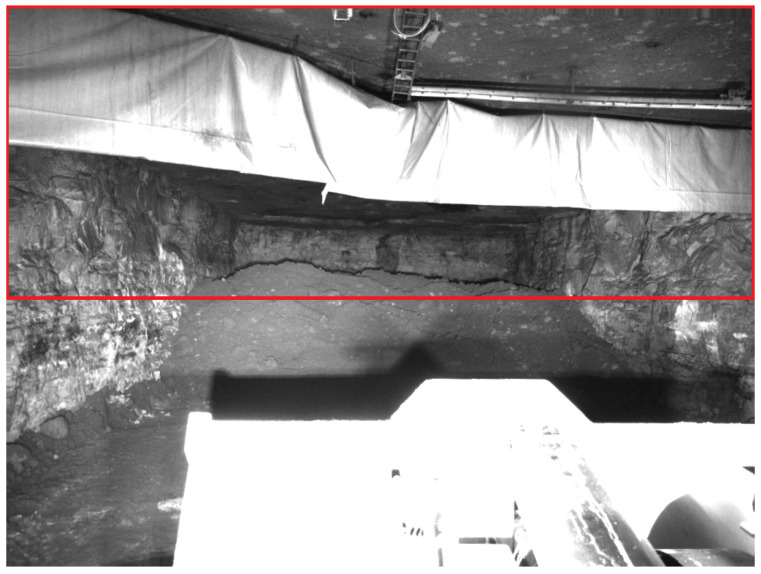
Example image taken from the left camera. The red rectangle shows the area that is set to half intensity on average by controlling the exposure time and the gain. The bright bucket is overexposed due to this optimization, but the environment is easily recognizable.

Even with additional headlights, an underground mining environment will always be relatively dark and, therefore, challenging for cameras. To mitigate this problem, attention was paid to the optical parameters controlling the brightness of the images. In particular, exposure time and gain were considered for this aspect. Exposure time denotes the time during which the optical sensors collect light before they combine it into an image. Gain is an electronic amplification of the optical signal and will linearly increase the brightness of the final image. To adapt to changing light conditions during operation, both exposure time and gain were automatically controlled. Every third frame, these parameters were set so that the upper half of the image achieved, on average, half intensity. The parameters were set only according to the top half to avoid including the bucket that is illuminated by the headlights and thus much brighter than the environment. Including it in determining the average would lead to overall much darker images, in which the environment would be harder to recognize. Figure 3 shows an exemplary image in which the area used to determine image brightness is marked with a red rectangle.

As the cameras are freely mounted on a profile, the system has to be calibrated so that the exact optical parameters of the two cameras and their orientation with respect to one another are known. As long as the cameras stay fixed on the profile, the calibration data does not change. Both the calibration and stereo processing were performed using the Nerian SceneScanPro system, which also provides time synchronization between the cameras, so there will always be two corresponding images taken at the same time.

The first step is image rectification, during which radial distortions from the cameras are removed, and the two images are projected into a common image plane. Then comes the stereo matching itself; for each pixel in the left image, the corresponding pixel in the right image has to be determined. This problem is simplified due to the image rectification, as the corresponding pixels will be at the same height in both images. To solve the matching problem, a variation in the semi-global matching algorithm [14] is applied to the rectified images from both cameras. The important output of this algorithm is the disparity; that is, for every pixel in the left image, return the distance to the corresponding pixel in the right image. After that, several post-processing steps are applied to create a high-quality disparity map. These post-processing steps are shown in the following list:

- Consistency check: ensure that the stereo matching has the same result, whether it is applied left-to-right or right-to-left.
- Uniqueness check: ensure that the stereo matching has a single optimal solution.
- Gap interpolation: fill small gaps in the disparity map using linear interpolation.
- Noise reduction: remove outliers in the disparity map.
- Speckle filter: remove small, isolated patches with a similar disparity.

In Table 2, the different parameters of the cameras and the stereo processing are listed.

From the disparity map, based on the position of a pixel in the image and the known camera parameters, the position of the pixel in 3D space can be computed using the formula in Equation (1). By applying this formula to every pixel in the left image, a point cloud is created.

(1) $$\begin{array}{cc} x= & \frac{\left(u-c_{x}\right)\cdot b}{d}\\ y= & \frac{\left(v-c_{y}\right)\cdot b}{d}\\ z= & \frac{f\cdot b}{d} \end{array}$$

where the following applies:

${\left(x,y,z\right)}^{T}$ = position of point in 3D space;
${\left(u,v\right)}^{T}$ = image coordinates of pixel;
${\left(c_{x},c_{y}\right)}^{T}$ = image coordinates of principal point;
*b* = baseline distance between the two cameras;
*d* = disparity at the pixel position;
*f* = focal length.

### 3.2. Muck Pile Detection in a Point Cloud

The interface between the stereo camera system and the computer processing the muck pile detection algorithm was handled via the Robot Operation System (ROS 2 Version Jazzy Jalisco) [15]. The stereo camera system periodically provides the image from the left camera and a point cloud. The detection system continuously takes the newest data from the stereo camera (when new data is available) and runs the detection algorithm on it. This structure ensures that the detection algorithm will always work on the newest available data and that the system can work together smoothly, regardless if the time between two images from the cameras or the runtime of the detection algorithm is longer.

**Figure 4:**
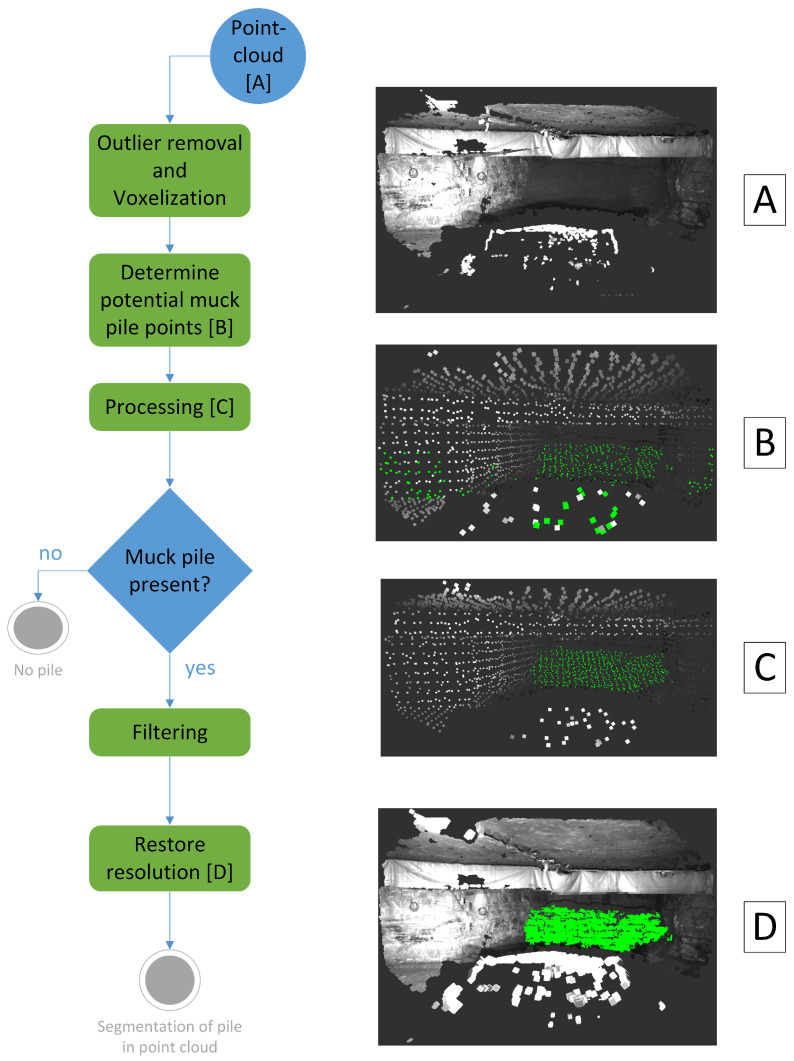
General overview of the operations that the proposed algorithm performs (**left**). Display of different stages during the segmentation (**right**).

A general overview of the muck pile detection algorithm is shown in Figure 4.

The original point cloud shown in Figure 4A is too large to be processed efficiently. So, as a first step, the point cloud is downsampled using voxelization. Outliers are removed from the downsampled point cloud by removing points that have fewer than n points in a radius, r, around them. In the next step, the surface normals for each point are computed by fitting a plane through the point and its nearest neighbors. The surface normals are used to determine candidate points, i.e., points that could potentially be part of a muck pile, as proposed by [10]. Due to the structure of the mine, the floor and roof surface normals have a large vertical and a small horizontal component, while the wall surface normals have a small vertical and a large horizontal component. The surface normals of the muck pile point diagonally upwards and have small diagonal components. Candidate points are selected on the basis of these characteristics. To be more precise, if the horizontal component of the normal vector, $n_{x}$, has an absolute value smaller than 0.3, and the absolute value of the vertical component of the normal vector, $n_{y}$, lies between 0.05 and 0.95, a point is considered a candidate for belonging to a pile.

The candidate points found in this way are marked in green in Figure 4B. The figure shows that there are points that are part of the muck pile that have not yet been classified as candidate points. In addition, there are points outside the muck pile that have been incorrectly marked as candidate points.

These points are considered in the following processing step. A KDTree is used to determine the number of candidate points in a neighborhood of each non-candidate point. If there are more than n candidate points in a neighborhood of a non-candidate point, this point becomes a candidate point. To remove the points incorrectly marked as candidate points, the candidate points are clustered. Only the biggest cluster remains part of the candidate points. The result of the processing step is displayed in Figure 4C.

In the following filtering step, different filters are applied to ensure that the detected cluster of candidate points belongs to a muck pile. For example, the slope of the muck pile is estimated by fitting a plane through it using the Random Sample Consensus (RANSAC) algorithm. If it is too steep, it is assumed to indicate either a wall or a hanging muck pile where it would be too dangerous to load from. In the context of this work, no muck pile will be detected in both cases. In later work, it might make sense to further differentiate these two cases.

Another filter concerns the distance to the muck pile. This is estimated via the depth dimension of the closest point in the detected muck pile. Euclidean distance does not make much sense, as the actual reference should not be the single point where the camera is but the LHD as a whole, which is several meters wide, so the additional computational cost to compute it does not pay off for this filter. We define both a minimum and a maximum distance to the muck pile. The reasoning behind the minimal distance is that, beyond it, the stereo matching becomes less stable, and the geometric structure is harder to detect, as the shovel will occlude most of the muck pile, so the detection result will be unreliable. Beyond that, if we are actually that close to the muck pile, the detection result is not needed anymore, as there is not enough room to maneuver. The reasoning behind the maximal distance is that the detection results also become less reliable beyond a critical distance. While it is good to provide a result as soon as possible, in the current system, the LHD navigation will work based on the first detection and not accept any updates, so the first result should already yield a good segmentation of the muck pile. To assure that, results beyond a maximal distance are discarded to wait for a better view of the muck pile. Still, a relatively large maximum distance was chosen. Upon consultation with Epiroc, which handled the attack point calculation based on the muck pile segmentation, it was determined that an earlier, rough outline of the muck pile would be more useful than a later, better segmentation of it.

When corners are driven around, the muck pile will become visible bit by bit instead of all at once. It is important that only complete muck piles are sent to the LHD. Otherwise, the draw point cannot be calculated reliably. Two filters were implemented to solve this problem. The first filter checks whether the width of the muck pile candidate is in a certain range. The second filter checks whether the muck pile candidate is roughly centered in the image on the horizontal axis. Only if both filters pass will the candidate points be recognized as a muck pile.

As a final prevention against false positives due to noise, the result is sent to the LHD only after a muck pile is detected in three consecutive frames.

In Table 3, the different parameters of the muck pile detection algorithm and their experimentally obtained values are collected. The computations on the point cloud were performed using the Open3D library (Version 0.19.0) [16].

If the cluster of candidate points passes all the filters, it is considered a muck pile. In this case, the original resolution is restored. To achieve this, a voxel grid is formed around the detected cluster points. All points of the original point cloud that lie within this voxel grid are then selected as muck pile points. The result of this operation is displayed in Figure 4D. This information is then sent to the system in the LHD, which will compute the optimal attack point based on this data.

Communication with the LHD takes place with the help of gRPC (Version 1.73.0) [17]. gRPC is a framework for the communication between devices based on remote procedure calls. When the LHD is approaching the stope from which it is about to load material, the loading process starts. From that point on, periodic queries are sent to the muck pile detection system, whether a muck pile can currently be detected or not, which is answered with a yes-or-no answer. This answer is based on the last completed detection to keep the time delay between query and answer as short as possible. If a muck pile is detected, a different query for its points is sent, which is answered with the detected muck pile points corresponding to the previous successful detection. These are then used for further processing beyond the scope of this work. Once a muck pile segmentation is sent, no further queries will be sent in the current loading process. This makes it important to only send a result when a segmentation of sufficiently high quality has been obtained. Only when the next loading process starts will the pile detection system start again to produce a fresh segmentation of the pile.

Another application of the gRPC interface was establishing time synchronization between the different systems. The computations took about 250 ms for a point cloud, which reduces the effective frame rate of the complete processing pipeline to 4 Hz and introduces a delay between the moment the cameras take an image and the moment a corresponding pile segmentation reaches the internal system in the LHD. During this delay, the LHD is moving and thus changing its position and orientation. Preliminary tests showed that, while the change in position is negligible, the change in orientation is not. When all systems are time-synchronized, the delay can easily be computed, and therefore, the discrepancy due to this delay can be compensated. This delay could be reduced with higher-grade hardware, including using a GPU for the point-cloud computations. However, as the successful experiments show, the system already works well with the current setup, which has a low power consumption due to the lower-grade hardware.

## 4. Test Setup and Results

The muck pile detection system was tested in two different underground mines. The first mine is the Kvarntorp mine near Örebro in Sweden. It is a former room-and-pillar limestone mine. Today, it is no longer in production but is used as a test mine by Epiroc. The second mine is the Kittilä mine in northern Finland operated by Agnico Eagle [18]. The Kittilä mine is the largest active gold mine in Europe, producing about 230,000 oz of gold per year using the cut-and-fill method.

In neither mine were external light sources employed at the pile, as they are unrealistic at an active stope.

**Figure 5:**
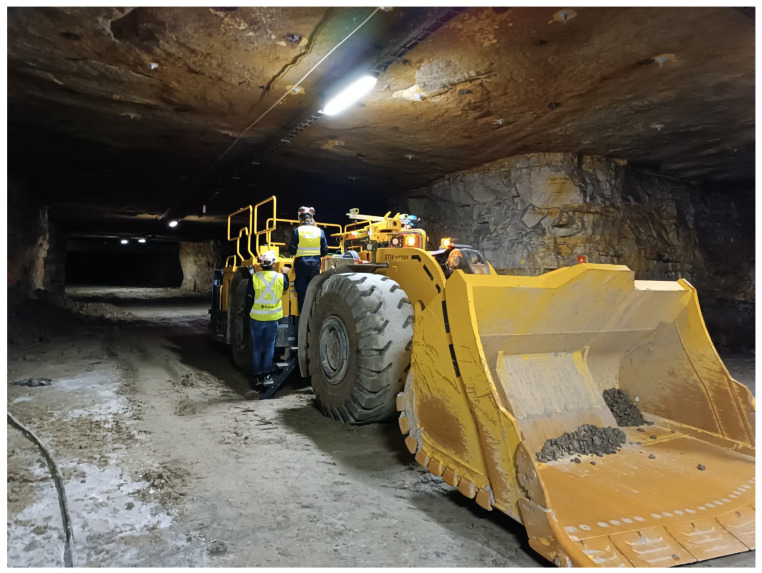
ST14 used for testing the muck pile detection system.

For the tests and demonstration of the system, an Epiroc Scooptram ST14 (Epiroc, Stockholm, Sweden) was used. It has a loading capacity of 14 t [19]. An image of the machine is shown in Figure 5. The muck pile detection system was integrated into a larger loading system, enabling the machine to complete the whole process autonomously without a driver while being supervised remotely.

At first, isolated tests of the stereo camera were performed in Kvarntorp to make sure it could accomplish the task. These tests provided insight into the specific mechanical requirements of the installation and also data that was used to tune the optical parameters and the parameters of the detection algorithm.

### 4.1. Integration Test in Kvarntorp Mine

**Figure 6:**
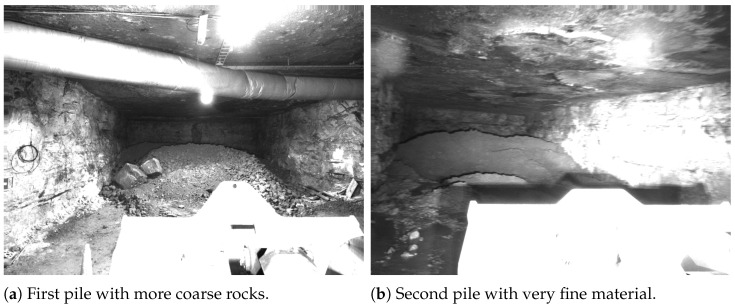
The two piles in the Kvarntorp mine on which automated loading was tested.

As the next step, integration tests were performed in Kvarntorp to make sure the system works in combination with the attack point analyzer to provide the necessary data for automatic loading. During the final integration test, 44 automatic loading attempts were performed on two different muck piles. The first pile was 11.4 m wide. Its stope could be entered in a straight line or around a curve, both of which were tested. The second pile was 10.7 m wide. Its stope could only enter around a curve. This pile was more challenging, as, due to repeated loading and unloading, the material became very fine. Since it also had high water content, in combination, it looked mostly like mud. This yielded less optical contrast inside the pile than would be expected from fragmented rock, which made the stereo matching more challenging. Figure 6 shows a side-by-side comparison of the two piles.

Sixty-one percent of the trials were successful. Thirty percent failed due to navigation problems unrelated to the muck pile detection system. This includes failures to enter the stope, failure to successfully navigate to an attack point, and failure to exit the stope after the shovel was filled. Nine percent failed because, after the 36th trial, one of the cameras was hit with dirt, which disrupted the stereo matching. This happened only once, but it caused four trials to fail before the problem was identified and solved.

In the trials where a bucket could be filled, the amount was estimated both by volume optically and by tonnage using an integrated scale. While the tonnage could be measured much more accurately, it was systematically biased: the bucket was not large enough to accommodate the relatively low density of the material, so, in many test runs, the nominal load was far from being reached, although the bucket was already full. On average, over all applicable trials, the bucket was filled to 94% and carried a mass of 9.3 t.

**Figure 7:**
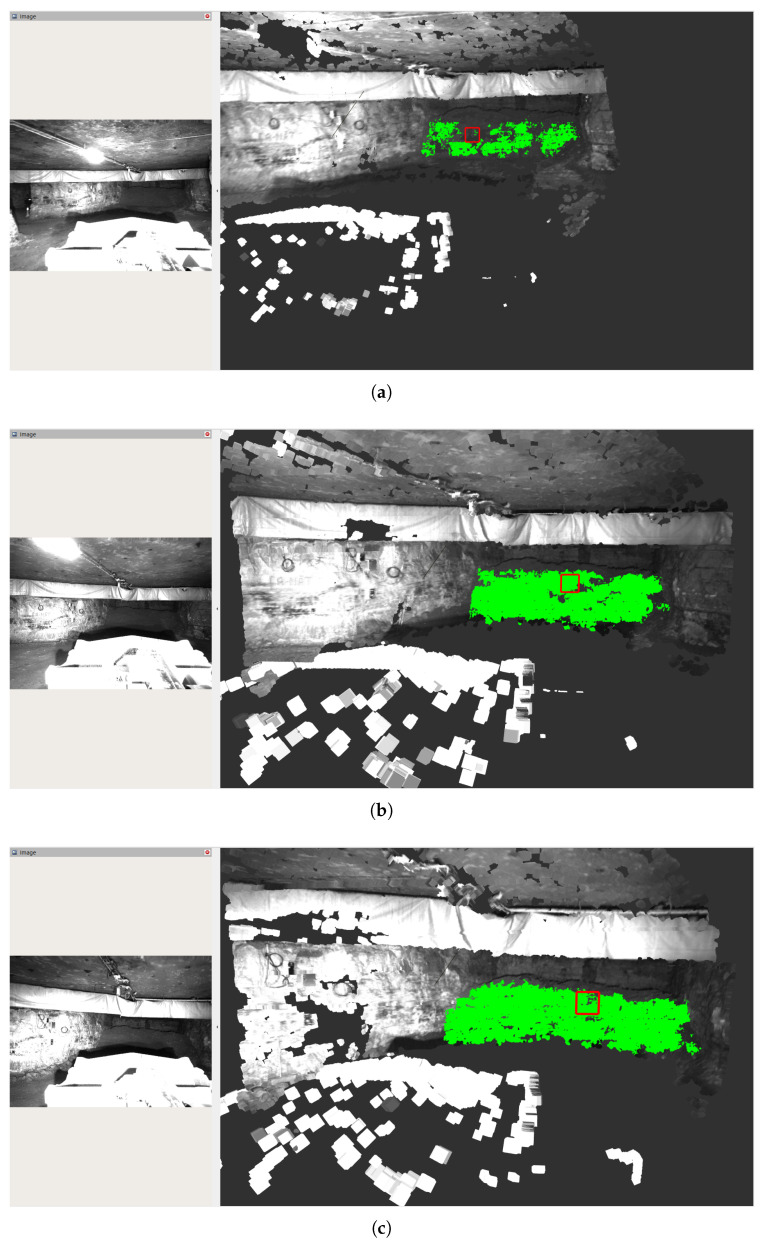
Muck pile detection results from a single loading sequence at a distance of 30 m (**a**), 25 m (**b**) and 20 m (**c**) from the camera respectively. On the left, the image from the left camera is shown, while the right shows the computed point cloud using the color information from the left camera with the detected muck pile points marked in green. The red square has a size of 1×1 m to illustrate the scale. The pile is 12.1 m wide and 3.8 m high. It was estimated as 12.5 m wide and 2.92 m high (**a**), 12.1 m wide and 3.46 m high (**b**) and 12.7 m wide and 3.42 m high (**c**) respectively. Even though this sequence represents a challenging case where the stereo matching suffers under low optical contrast in the pile, relatively good segmentation can still be achieved at a distance of 25 m from the camera.

Illustrative examples of the muck pile detection results from a single test run are shown in Figure 7. A rough outline of the muck pile is already detected at a distance of 30 m from the camera. The quality of the segmentation greatly improves at a distance of 25 m, which still leaves time to maneuver to an attack point.

The relationship between the distance to the muck pile and the time until the LHD will collide with the muck pile is illustrated with concrete values in Table 4. These results are obtained by varying the maximum distance parameter described in Section 3.2 in recordings of different loading sequences. This includes three loading sequences where the LHD entered the stope around a curve relatively close to the pile and five loading sequences where the LHD approached the pile in a straight line for more than 30 m. After each loading sequence, the loaded material was dumped back onto the respective pile, so it changed its shape without getting smaller. It should be noted that these distances are computed from the stereo camera. The tip of the shovel is around 5 m in front of the cameras. A sharp difference between a straight approach to the pile and an approach around a curve is apparent, as, in the latter case, the LHD might already be relatively close to the pile when it is around the curve enough that the pile is in the field of view of the cameras.

In Table 5, the accuracy of the detection is evaluated via the estimated and true widths of the segmented pile at a distance of 25 m from the left camera. Again, the types of approach to a pile have to be differentiated: when approaching around a curve, the system often already detects and segments parts of the pile before it comes into view completely, which leads to vastly underestimating its width. With a straight approach, this problem does not occur, leading to much smaller errors.

Table 6 compares these accuracies to the ones reported in [10] (the mean relative error was manually computed from values in Table 6 of the cited work). This comparison should be taken with a grain of salt, as the two systems were tested in different environments (the former room-and-pillar Kvarntorp mine in the case of this work and a sublevel stoping mine in Chile in the case of [10]). The mine in Chile has much narrower tunnels with a pile width between 3 m and 3.4 m compared to 10.6 m to 12.0 m in Kvarntorp. It is not quite clear how far from the pile the LHD was during the measurements (or, rather, at what range of distances, as several measurements were integrated over time). It seems to be a relatively straight approach to the pile there. All in all, this comparison should be treated as only a rough estimate, as several parameters either significantly differ from each other or are at least unclear.

### 4.2. System Demonstration in Kittilä Mine

Finally, the complete system was demonstrated in front of the NEXGEN SIMS project consortium in the Kittilä mine. A video of this demonstration was published under https://www.youtube.com/watch?v=z9gNyciqV7Y (accessed on 30 April 2025).

The final demonstration of the autonomous material handling took place at a depth of 750 m within Agnico Eagle’s Kittilä gold mine, located in Finland. It included a fully autonomous material handling cycle utilizing a battery-electric LHD and mine truck from the manufacturer, Epiroc (Stockholm, Sweden). The demonstration was monitored from a nearby control room set up outside the Autonomous Operating Zone (AOZ), which was designated for the autonomous machine operation. The muck pile used for the demonstration of autonomous loading was about 11 m wide.

The cycle begins with the LHD autonomously entering the working face with the muck pile. As the LHD approaches the muck pile, it is detected via the installed stereo camera system. The detection happens while driving, so the information is used with internal software to determine the best attack point to enter. The LHD drives to the attack point to fill the bucket. After that, the material is hauled to the defined mine truck position. The LHD and the mine truck communicate with each other via a 5G network installed in the AOZ. As soon as the mine truck confirms that it is ready for loading, the bucket is unloaded into the mine truck. This process is repeated multiple times until the mine truck is filled. Every time the LHD returns to the muck pile, its shape has changed.

During this demonstration, the autonomous loading from the muck pile with a driverless LHD worked well, successfully proving the effectiveness of the complete system, including the component for muck pile detection. During preparation for this demonstration, the parameters optimized in the Kvarntorp mine still worked well in this new environment, indicating the robustness of the detection algorithm to different underground environments.

### 4.3. Limitations

An important limitation of the sensor setup at the current stage is that the cameras are missing a cleaning mechanism against dust and dirt hitting their case. As the whole system depends on matching two camera images, the quality is reduced drastically if parts of one image are blocked out due to dust. It might also make sense to try to at least detect the problem algorithmically, as the current system will just output nonsensical results in that case. During the experiments, it was fortunately only a minor problem, as the cameras on top of and in the middle of the LHD were not hit with much dust or dirt, but this cannot be guaranteed in a production environment.

Neither mine in which this system was tested featured large amounts of airborne dust, which would limit the visibility range of the cameras. Before the muck pile system is employed in such an environment, further experiments would be necessary to estimate the influence of airborne dust on the system.

An additional limitation is that the algorithm expects the muck pile to be consecutive and removes any parts not connected to the main muck pile during the clustering step described in Section 3.2 as noise. While it is important not to have such noise in the detection, this means that smaller muck piles not directly connected to the main muck pile will be discarded, although they might be useful to consider when deciding how to approach the muck pile, as they may represent material lying apart from the main muck pile.

## 5. Conclusions and Future Work

### 5.1. Conclusions

This work has presented a novel system for detecting muck piles in underground mining environments using a stereo camera. A 3D representation of the environment was constructed by processing the stereo images. Using geometric conditions, a muck pile could then be detected and segmented. Based on this segmentation, autonomous loading from a muck pile could be performed. This proves that a stereo camera is indeed a viable sensor to provide the three-dimensional data necessary for material loading with an LHD. The muck pile detection inside a point cloud from the stereo camera was performed using a geometry-based algorithm.

It was demonstrated to work reliably in two different mines as a component integrated into an autonomous loading system. The detection system both is robust against motion blur and can compute results fast enough so that the muck pile detection works from an LHD driving at regular speed. This means no time is lost on the LHD slowing down or stopping. Therefore, the system can be efficiently integrated into existing processes without increasing the cycle time.

### 5.2. Future Work

For long-term deployment in a production environment, a wiper should be added to the cameras to remove dust and dirt from them. It might also make sense to add a dirt detection routine by looking for portions of the image that are completely black over several frames.

The algorithm could be extended to cover more variants in the environment. One example from the experiments is smaller, unconnected muck piles in the same stope as the main muck pile. When the system is applied in a production setting over a longer period of time, more unforeseen situations like this will probably occur. Incorporating them into the algorithm would make it more robust and allow for a higher level of automation. In any case, it is critical to be aware of the assumptions behind the systems to recognize where it can be applied reliably.

Finally, there are still important issues beyond the scope of this work. For example, if a large enough boulder is in the muck pile, it needs to be recognized as such, as it requires special treatment and cannot just be loaded normally together with the surrounding rock. To achieve autonomous driving, the sensor systems on the machine also need to provide obstacle detection to prevent accidents.

## Figures and Tables

**Table 1:** Previous works on muck pile detection in mining environments.

Title	Author	Year	Sensor Technology	Environment
Stereo vision in LHD automation [6]	Mark Whitehorn, Tyrone Vincent, Christian Debrunner, and John Steele	2003	Stereo camera	Edgar Experimental Mine
Field test of autonomous loading operation by wheel loader [7]	Shigeru Sarata, Noriho Koyachi, and Kazuhiro Sugawara	2008	Stereo camera, laser range finder	Free-standing crushed sandstone pile above ground in a field test site in Tsukuba
Consistent pile-shape quantification for autonomous wheel loaders [8]	Martin Magnusson and Håkan Almqvist	2011	Actuated lidar	Gravel pile above ground
Continuous control of an underground loader using deep reinforcement learning [9]	Sofi Backman, Daniel Lindmark, Kenneth Bodin, Martin Servin, Joakim Mörk, and Håkan Löfgren	2021	Depth camera	Simulated narrow underground drift with muck pile
Autonomous Loading System for Load-Haul-Dump (LHD) Machines Used in Underground Mining [10]	Carlos Tampier, Mauricio Mascaró, and Javier Ruiz-Del-solar	2021	2D lidars	Sublevel stoping mine
Autonomous detection and loading of ore piles with load–haul–dump machines in room-and-pillar mines [11]	Daniel Cardenas, Patricio Loncomilla, Felipe Inostroza, Isao Parra-Tsunekawa, and Javier Ruiz-Del-solar	2023	2D and 3D lidars	Werra Potash Mine (room and pillar)
Autonomous Mining Vehicle Control [4,12]	Jasu Jari and Siivonen Lauri	2023	Lidars	Mines with narrow drifts and a fixed drawpoint

**Table 2:** Relevant parameters of the camera setup. These include (**a**) the optical parameters of the cameras and (**b**) the parameters of the stereo matching.

Parameter	Value
(**a**) Optical Parameters	
Resolution	1024×768 pixels
Focal length	6 mm
Focal ratio	f/2.4
Framerate	32 Hz
Exposure time	Set automatically below 30 ms
Gain	Set automatically below 17 dB
(**b**) Stereo Parameters	
Baseline length	1 m
Maximal disparity	256 Pixels
Resolution of disparities	1/16th pixel
Penalty for disparity change on image edge	3
Penalty for disparity change without image edge	14
Penalty for disparity discontinuity on image edge	22
Penalty for disparity discontinuity without image edge	65

**Table 3:** Parameters of the muck pile detection algorithm.

Parameter	Value
Voxel size for downsampling	0.5 m
Outlier removal	Min. 10 neighboring points in a radius of 1 m
Normal computation neighbors	10 nearest neighbors considered
Normal criteria for muck pile	$|n_{x}|<0.3$ and $0.05<|n_{y}|<0.95$
Neighborhood criteria for muck pile	5 points in a radius of 1 m belong to pile
Minimum muck pile width	5 m
Minimum distance from cameras to muck pile	5 m
Maximum distance from cameras to muck pile	30 m

**Table 4:** Average time from the moment of successful muck pile detection until collision of the LHD with the muck pile for different minimum distances at which the detection is considered. For these averages, five loading sequences in which the pile was approached in a straight line and three loading sequences in which the pile was approached from a curve were examined. In an approach around a curve, more time passes before the pile comes into the view of the cameras, and by then, it is often already relatively close. That is why the overall times are lower, and there is only a slight relationship to the minimum distance.

Minimum Distance from Stereo Camera to Muck Pile	Average Time Until Muck Pile Collision (Straight Line)	Average Time Until Muck Pile Collision (Curve)
30 m	14.5 s	6.1 s
25 m	11.7 s	6.1 s
20 m	8.6 s	5.5 s

**Table 5:** The accuracy of the estimated pile width for different loading cycles. The accuracy is a lot lower when the pile is approached around a curve, as in that case, segmentation will already be sent before the pile is in full view.

Approach	True Pile Width	Estimated Pile Width	Absolute Error	Relative Error
Straight	11.5 m	11.2 m	0.3 m	3%
Curve	10.6 m	4.94 m	5.66 m	53%
Curve	10.6 m	6.79 m	3.81 m	36%
Curve	10.5 m	5.75 m	4.75 m	45%
Straight	11.9 m	12.4 m	0.5 m	4%
Straight	12.0 m	12.2 m	0.2 m	2%
Straight	11.5 m	12.6 m	1.1 m	10%
Straight	11.5 m	11.8 m	0.3 m	3%

**Table 6:** Comparison of the accuracy of the pile detection in [10] and in this work. Ref. [10] is probably more comparable to a straight approach in this work, although the conditions still differ significantly. The lowest error is marked with italics.

Work	Mean Absolute Error	Mean Relative Error
Autonomous Loading System for Load-Haul-Dump (LHD) Machines Used in Underground Mining [10]	0.58 m	18%
Stereo vision-based underground muck pile detection for autonomous LHD bucket loading (straight approach)	*0.48 m*	*4%*
Stereo vision-based underground muck pile detection for autonomous LHD bucket loading (curved approach)	4.7 m	45%

## Data Availability

Restrictions apply to the availability of these data. Data were obtained from mines of Epiroc and Agnico Eagle Mines Ltd. and are available from the authors with the permission of the respective mine owner.
